# Human cytomegalovirus immediate-early protein promotes survival of glioma cells through interacting and acetylating ATF5

**DOI:** 10.18632/oncotarget.17150

**Published:** 2017-04-17

**Authors:** Ming Hu, Bin Wang, Dongmeng Qian, Mengyuan Wang, Rui Huang, Li Wei, Ling Li, Li Zhang, David X. Liu

**Affiliations:** ^1^ Department of Basic Medical Sciences, Qingdao University, Qingdao, China; ^2^ College of Life Sciences, Qingdao University, Qingdao, China; ^3^ The Hospital of People's Liberation Army, Weifang, China; ^4^ Department of Pharmaceutical Sciences, Washington State University College of Pharmacy, Spokane, WA, USA

**Keywords:** HCMV, IE86, glioma, ATF5, acetylation

## Abstract

Human cytomegalovirus (HCMV), a widespread beta-herpes virus, infects a high percentage of gliomas. HCMV is specifically detected in human gliomas at a low level of expression raises the possibility that it may regulate the malignant phenotype in a chronic manner. Although HCMV is not recognized as an oncogenic virus, it might dysregulate signaling pathways involved in initiation and promotion of malignancy.

Here, our immunohistochemical staining reveals that nucleus staining of the HCMV 86-kDa immediate-early protein (IE86) is markedly increased in GBM (58.56%) compared with that in nontumorous samples (4.20%) and low-grade glioma(19.56%). IE86 staining positively correlates with the staining of activating transcription factor 5 (ATF5) which is essential for glioma cell viability and proliferation suggesting that HCMV IE86 could have important implications in glioma biology. Moreover, we find that the IE86 overexpression enhances glioma cell's growth *in vitro* and *in vivo*. We demonstrate that IE86 protein physically interacts with, and acetylates ATF5 thereby promoting glioma cell survival. Therefore, our findings illustrate the biological significance of HCMV infection in accelerating glioma progression, and provide novel evidence that HCMV infection may serve as a therapeutic target in human glioma.

## INTRODUCTION

Malignant gliomas which character as invasive, aggressive, and neurologically destructive are considered one of the deadly human cancers. Despite current surgical resection followed by a combination of radiotherapy or chemotherapy is feasible, the median survival is only about 1 year [1]. Similar to a class four diffuse astrocytoma, Glioblastoma multiforme (GBM) consists generally of neoplastic astrocytes which is the most abounding type of glia, but neoplasm specimens as well include additional nonneoplastic cell types, including oligodendrocytes, neurons, macrophages, glial and nervous stem cells. A developing number of issues indicate that these tumors arise from neuronal stem cells [2–5]. Our previous work has demonstrated that neural stem cells are permissive to human cytomegalovirus (HCMV) infection, resulting in abnormal differentiation or inhibition of differentiation into normal astrocytes [6]. This result was consistent with previously published studies [7, 8]. HCMV infection also induced glioma cancer stem cells (GCSCs) phenotypes which express the same marker CD133 with neural stem cells. HCMV infection triggered increasing expression of CD133 and other GSCS markers (Sox2, Notch1, Nestin, Oct4) and inhibited GCSCs differentiation [9]. Interestingly, several reports have confirmed that HCMV viral genes and proteins expression were detected in most GBM cells but not in surrounding normal brain, or other neuropathologies, indicating a potential association between gliomas and HCMV infection [10–12].

HCMV belongs to the herpesvirus family causing a widespread and persistent infection in human population. The frequency of infection that leads to a life-long persistence ranges from 40% to 90% in adult population [13, 14]. Brain is the preferential site of HCMV infection resulting in sequelae such as mental disorder or epilepsy as functional disorders [15]. Cobbs was the beginning group to report the connection between HCMV infection and malignant gliomas. They reported that virus genes and proteins were detected in high-grade and low-grade gliomas with a high percentage [16]. Since then several groups reported a potential association between HCMV infection and gliomas. Oncogenic DNA viruses like Epstein-Barr virus (EBV), hepatitis B virus (HBV), and human papilloma virus (HPV) which own the tumor antigen can establish chronic viral infections. Unlike HBV infection resulting in hepatoma or EBV in nasopharyngeal carcinoma, HCMV has not been concerned in transforming human cells. Even so, growing reports that HCMV is specifically expressed in human malignancies raises the possibility that HCMV could favor tumor progression without being an oncogenic virus. The accurate function of HCMV in gliomas is under investigating. HCMV could modulate the tumor malignant phenotype through affecting the cell survival, cell cycle and invasive potential. Oncomodulation means that HCMV might infect tumor cells and increase their malignant while not affecting transformation directly. Tumor cells may provide genetic surroundings like transcription factors or signaling pathways, which enables HCMV to maintain its oncomodulatory potential, while it can't be manifested in normal cells. HCMV induced oncomodulation could activate regulatory proteins, which influence cells proliferation, survival and a more malignant tumor phenotype.

The first viral genes to become active after HCMV infection are the immediate-early genes. The most abundantly expressed products of this first set of genes are termed the immediate-early 1 and 2 proteins (IE72 and IE86). These IE proteins are trans-activators of viral as well as cellular gene expression. The IE86 protein is a strong trans-activator that interacts with factors of the basal-transcription machinery [17]. The IE86 protein involved in transactivation of viral and cellular promoters is unique among viral regulatory proteins because it could regulate viral and cellular promoters negatively and positively [18]. IE86 also does form complexes with p300 and the CREB-binding protein (CBP), which in turn serve as adaptor proteins for CREB function [19].

Viruses are intracellular parasites that rely on hosts to provide environment to complete their life cycle. As a means to accomplish this, viruses may modulate host transcription factors to make sure the infected cell does not die. Activating transcription factor 5 (ATF5) is a member of ATF/CREB family with basic leucine zipper (bZIP) domains. Because of the leucine zipper motif and basic region enriched with lysine and arginine residues, ATF5 is both involved in DNA binding and protein-protein interactions [20, 21]. ATF5 expression have been detected in brain tumors, and is particularly high in malignant glioma, while not been detected in mature astrocytes and brain neurons [22, 23]. Disturbance on ATF5 function or expression drives apoptosis of glioma cell lines while not affecting survival of nonneoplastic brain cells [24, 25]. The transcriptional activity of ATF5 is modulated in part by its post-translational acetylation. The binding of ATF5 to the coactivators p300 which have histone acetyltransferase (HAT) activity results in synergistic enhancement of ATF5 transactivation activity [26, 27]. The IE protein and ATF5 all express in glioma tissue and cell line and both of them can form complexes with p300. However, the oncomodulation mechanisms of IE through are largely unknown.

Here we report the relationship among HCMV infection, glioma classification and ATF5 expression in paraffin sections of surgically excised glioma tumors. To study HCMV infection effects on tumor, we established HCMV-infected and IE86 overexpressed glioma cell lines. The interaction between ATF5 and IE86 was confirmed by CoIP and Confocal. Nude mice models bearing human glioma cells were established to investigate the influence on tumor growth by HCMV IE86 overexpression. Together, our observations reveal a role for HCMV IE86 in regulation of ATF5 activity.

## RESULTS

### Expression of ATF5 and IE in human glioma sections specimens

**Figure 1:**
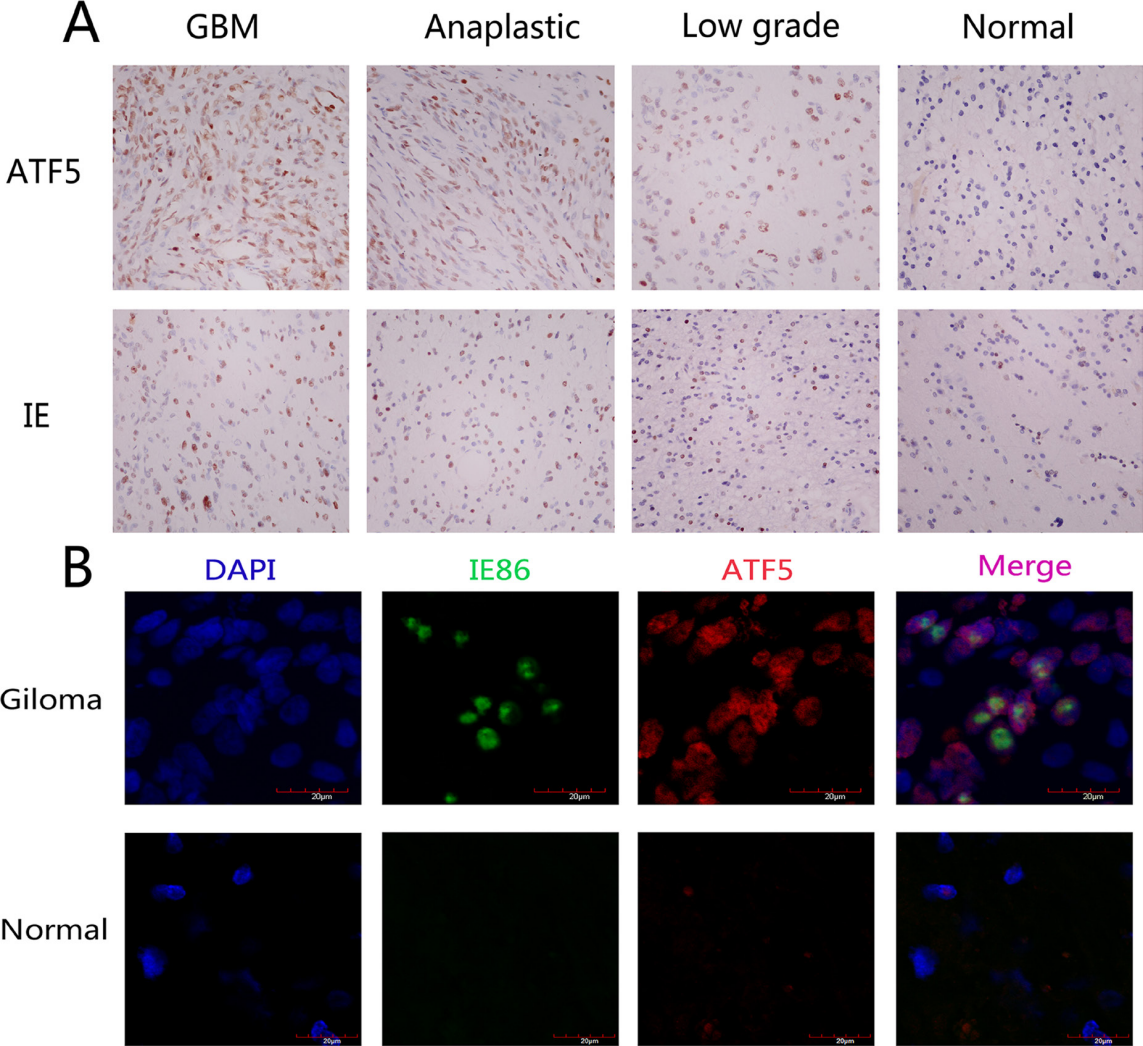
Expression of HCMV IE and ATF5 in normal brain tissue and human glioma samples (**A**) Representative photos of different grade glioma and hyperplasia tissues indicate the pattern of ATF5 and IE expression. The immunostaining for both ATF5 and IE are more uniform and intense in GBM when compared to the LGG and comparable hyperplasia tissues. Magnification: 400×. (**B**) Colocalization of ATF5 and IE in glioblastoma tissues and hyperplasia tissues. Frozen sections of glioblastomas and hyperplasia tissues were processed for triple immunofluorescence staining for IE (green), ATF5(red), and nuclei (DAPI; blue). These representative photos of glioblastoma and hyperplasia tissues indicate that cells in glioblastoma tissues displayed strong positive nuclei staining of ATF5 and IE but cells in hyperplasia displayed weak. Merged photos indicated that IE and ATF5 colocalized in the cell nuclei.

To confirm the existence of HCMV in gliomas and determine if there is a relationship between ATF5 expression and HCMV infection, 58 paraffin-embedded glioma sections were performed for immunohistochemical staining. We observed immunoreactivity of HCMV IE86 protein in all 25 GBMs, 10 anaplastic gliomas, and 18 low-grade gliomas, but seldom in the nonneoplastic brain tissue. We found IE86 positive reactivity in the GBM tumor cells nuclei and partly in the perinuclear cytoplasm (Figure 1A). We quantified the immunohistochemical staining of IE86. The overall positive rate of GBM samples was 58.56 ± 9.72, while anaplastic and low-grade tumors were 44.10 ± 21.27 and 19.56 ± 9.86 positive, respectively (Table 1). A very low level of IE86 expression was observed in control tissues compared with glioma (4.2 ± 2.77). In general, there was a statistically significant difference in IE86 expression between glioma samples and nontumorous samples. Next, we examined ATF5 expression in various kinds of the glioma specimens. Similar to the pattern we observed for IE86 expression, we found ATF5 protein high expression in tumor nucleus and cytoplasm of glioma tissues than normal tissues (Figure 1A). The overall ATF5 positive rate in GBM samples was 89.80 ± 2.97, while anaplastic, low-grade tumors and nontumorous samples were 86.90 ± 3.72, 29.22 ± 10.95 and 0.40 ± 0.894, respectively.

**Table 1:** Expression of ATF5 and IE proteins in tissues

Groups	samples	ATF5 (+)*	IE (+)^#^
	(*n*)	x¯ ± s	x¯ ± s
GBM	25	89.80 ± 2.97	58.56 ± 9.72
Anaplastic	10	86.90 ± 3.72	44.10 ± 21.27
LGG	18	29.22 ± 10.95	19.56 ± 9.86
Hyperplasia	5	0.40 ± 0.894	4.2 ± 2.77

ATF5 and IE86 expression positively correlates with the degree of malignancy in gliomas (^#^*F* = 50.87, *P* < 0.05; *F = 482.24, *P* < 0.05). Importantly, IE86 staining positively correlated with the proliferative activity indicated by ATF5 staining (*P* < 0.05, r = 0.810, by Spearman's correlation coefficient). Figure 1A displays the positive expression of both ATF5 and IE86 from representative glioma tumors. These tumors had nuclear staining for ATF5 and IE antigens. Not all cells in a tumor were positive for IE, possibly reflecting variable infection in glioma cells. Colocalization of ATF5 and IE86 expression was confirmed by double immunoflurorescent assay (Figure 1B). Therefore, our results suggest that HCMV infection existed in glioma tissues, and there was a significantly higher positivity of HCMV IE86 in GBM when compared to the LGG (*P* < 0.05). Interestingly, ATF5 expression is colocalized and correlated with HCMV IE expression, indicating that ATF5 may play a crucial role in the malignant phenotype of gliomas increasing under HCMV infection.

### HCMV infection and IE overexpression enhance tumor survivability subject to serum deprivation

**Figure 2:**
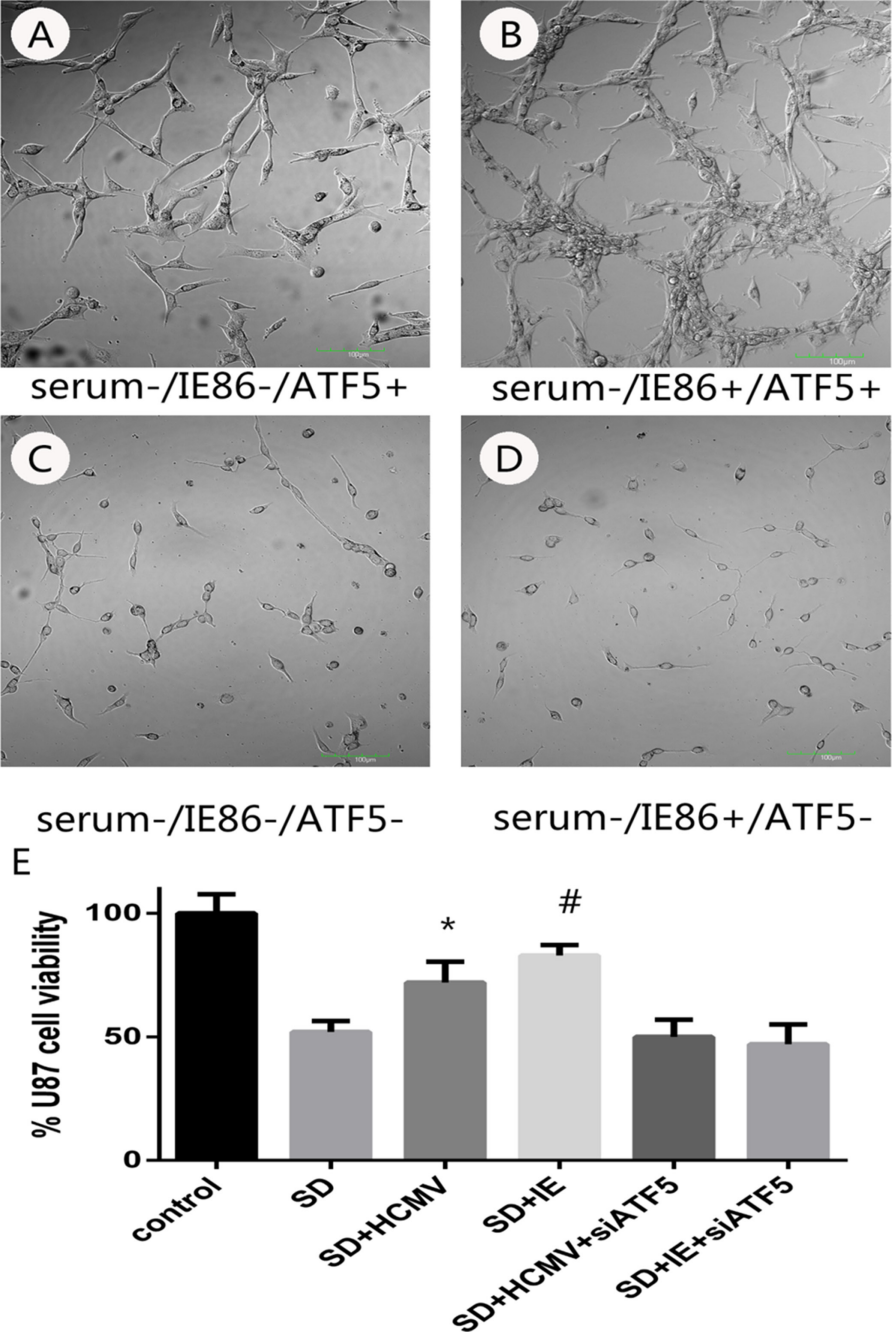
The effects of serum deprivation on survivability of U87 cells or RNAi-ATF5 U87 cells after HCMV infection or IE plasmids transfection (**A**–**D**) Morphological changes of U87 cells or RNAi-ATF5 U87 cells with serum deprivation or serum deprivation combined with IE86 overexpression. (**E**) MTT analyses of HCMV infection or IE plasmids transfection on U87cells and RNAi-ATF5 U87 cells after serum deprivation. *P < 0.05 vs SD, ^#^*P* < 0.05 vs SD.

**Figure 3:**
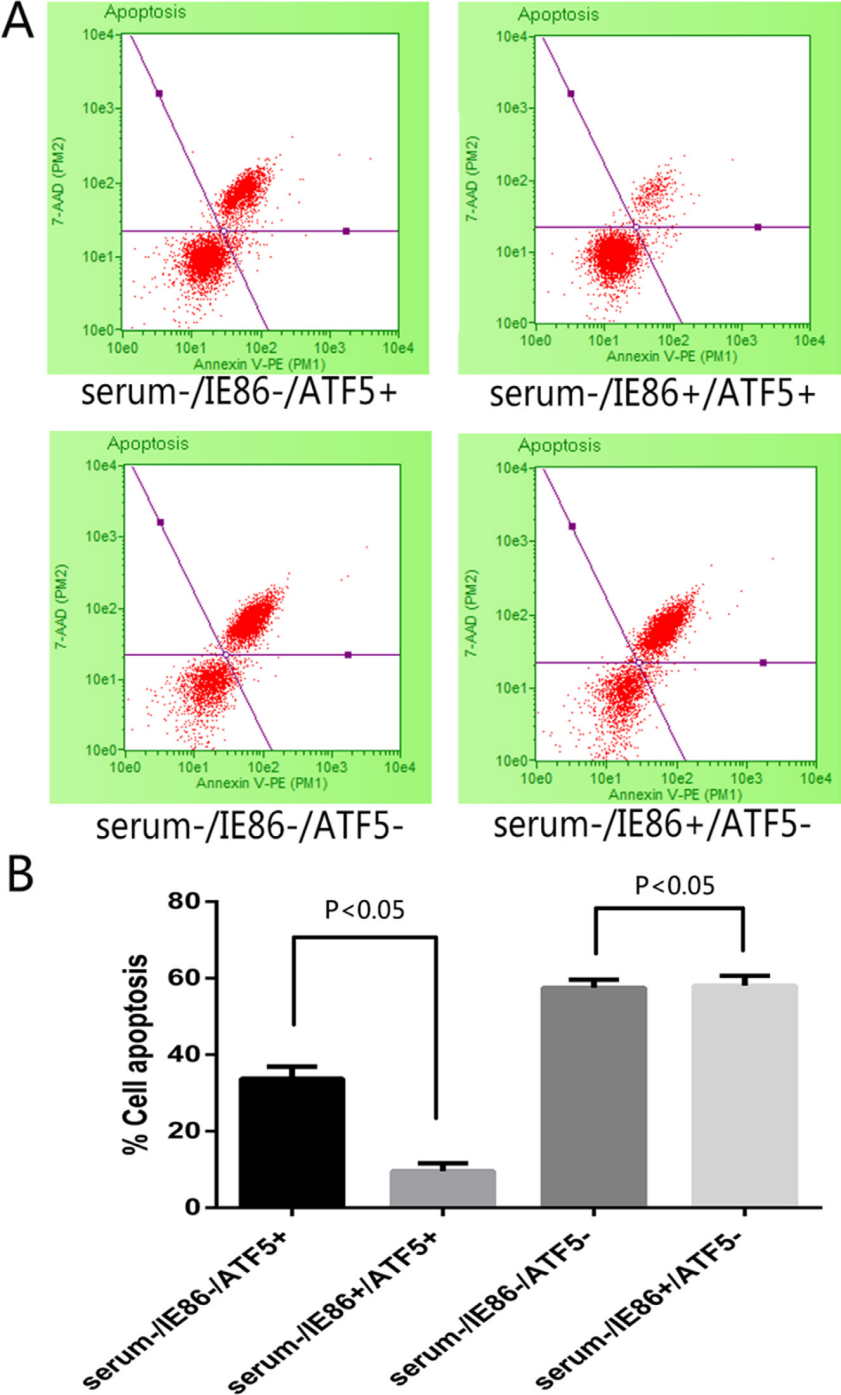
Apoptosis detection following treatment with serum deprivation and IE86 expression in ATF5 (+/ −**) U87 cells**. (**A**) Apoptosis measured by Annexin V/7-AAD staining with flow cytometry after serum deprivation 72 h. Events in each of the four quadrants are as follows: Lower-left: viable cells. Lower-right: cells in the early to mid-stages of apoptosis. Upper-right: cells in the late stages of apoptosis. Upper-left: mostly nuclear debris. (**B**) The total apoptosis rate obtained by Annexin V/7-AAD staining after 72 h treatment with serum deprivation in different cell group. IE86-expressing cells displayed significant resistance to cell apoptosis evoked by serum deprivation, but in RNAi-ATF5 U87 cells IE could not rescue the cells from apoptosis.

In order to answer the negative stimulations such as viral infection, cells activate the apoptosis program to trigger self-destruction. On the other hand, virus needs to maintain cell viability until the maximal virus replication is essential. To determine the role of HCMV infection on survivability of U87 glioblastoma cells, cells were infected with HCMV of MOI 10 or transfected with HCMV IE86 plasmid (Supplementary Figure 1). After 12 h cells were subjected to serum deprivation (SD) treatment that leads to cell death. Cell proliferation and apoptosis were detected in 72 h after SD. SD treatment evoked morphological changes (Figure 2A–2D) proliferation inhibition (Figure 2E) and apoptosis (Figure 3) in U87 cells, whereas HCMV IE86 overexpression cells displayed significant resistance to cell death (Figure 3).

Our previous work has demonstrated that ATF5 is a key factor for cancer-specific cell survival [28, 29] and it is highly expressed in U87 cell. And overexpression of ATF5 suppressed apoptosis in C6 and MCF-7 cells induced by serum deprivation [30]. In order to elucidate the molecular mechanisms for apoptosis resistance of HCMV or IE86, we next examined if the apoptosis resistance is related to ATF5. ATF5 is highly expressed in U87 cell, so a shRNA delivering by lentivirus vector was used to inhibit the expression of ATF5 in U87 cells (Supplementary Figure 2). In this condition, IE86 overexpression (Supplementary Figure 2) could not rescue the cells from apoptosis induced by apoptotic stimulation (Figure 2C and 2D). In addition, U87 cells transfected with d/n ATF5 which has the Pro-rich domain deleted and acts as dominant-negative [28] were also failed to be rescued by IE86 overexpression from the apoptosis (data not show).

### IE86 binds to ATF5 and acetylates ATF5 at K29 through P300

**Figure 4:**
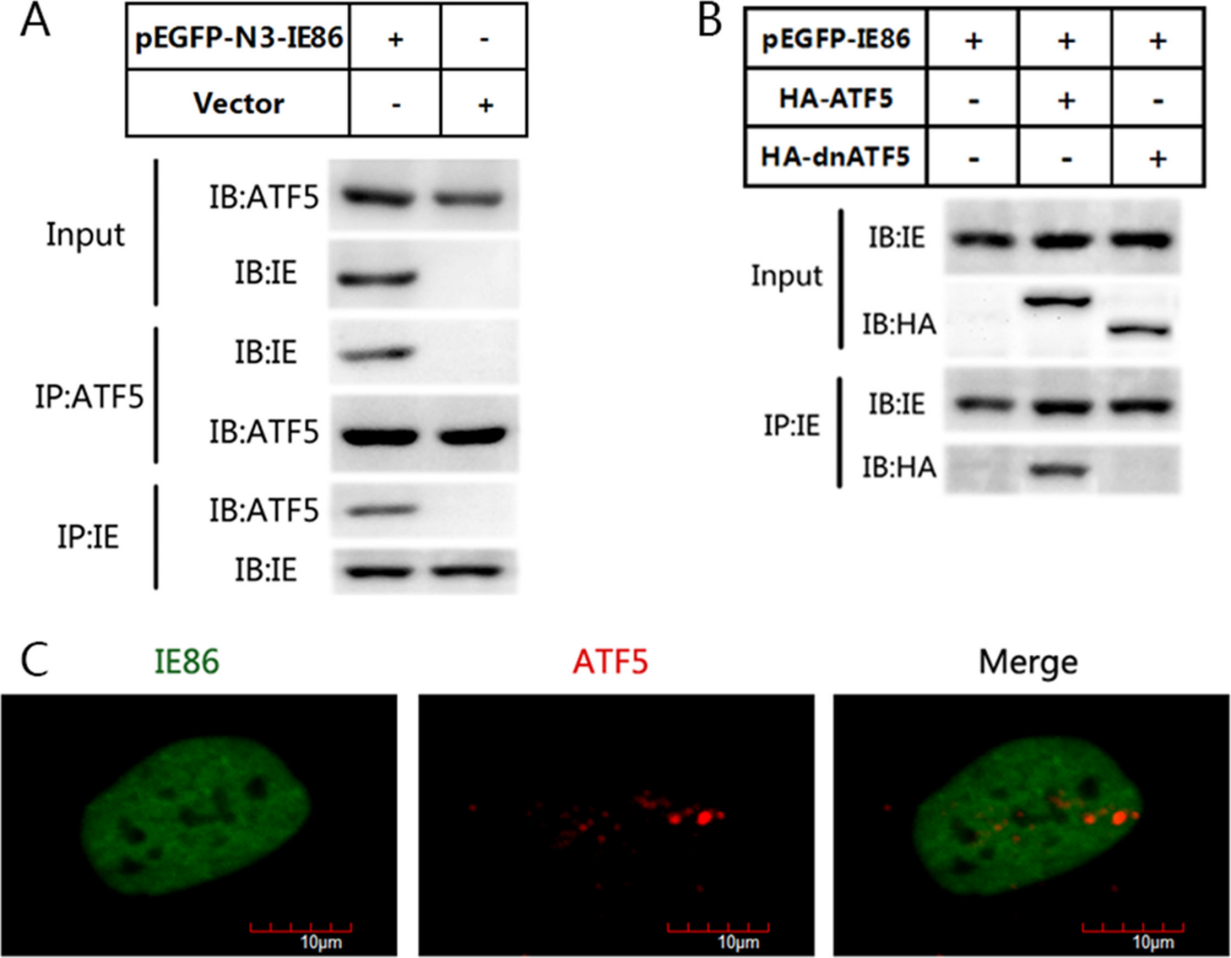
IE86 interacts with endogenous ATF5 but not with dnATF5 (**A**) U87 cells were transfected with or without IE86 constructs. Immunoblotting analyses of immunoprecipitates using indicated antibodies were performed. (**B**) C6 glioma cells were transfected with IE86 constructs and transiently transfected with a vector expressing HA-ATF5 or one expressing dnATF5. Expression of IE86 and FLAG-HA-ATF5 were detected using antibodies against IE86 and HA as indicated. (**C**) Immunofluorescence staining of ATF5 (red) and IE86 (green) in U87 cells U87 cells were transfected with IE86 constructs, fixed at 48 h posttransfection, stained with antibodies, and visualized using an Olympus confocal microscope system. The images of IE86 (green), ATF5 (red), and the morphology were used to generate the composite images. The colocalization of the two proteins is in the nucleolus. Bar scale is 10 μm.

ATF5 expression is colocalized and correlated with HCMV IE expression in glioma tissues. And serum deprivation led to U87 cell apoptosis whereas HCMV infection or IE86 overexpression suppressed this apoptosis. As protein-protein interactions are increasingly recognized as a mean by which viral and cellular regulatory proteins stimulate gene expression, we asked whether IE86 could interact directly with ATF5 to suppress cells apoptosis. To further find out the mechanism underlying ATF5-dependent IE86 apoptosis rescue, we examined whether IE86 interacts with endogenous ATF5. Immunoblotting with an anti-ATF5 antibody for the IE86 immunoprecipitation from U87 cells showed that endogenous ATF5 was coprecipitated with IE86 (Figure 4A), while a control IgG failed to bring down either IE86 or ATF5. This indicates that viral IE86 protein can interact with ATF5. ATF5 has an N-terminal Pro-rich domain and a C-terminal bZIP region. To determine which part of ATF5 interacts with IE86, we performed immunoprecipitation analysis using C6 cells transfected with a construct expressing GFP-ATF5 or GFP-dnATF5. GFP-dnATF5 is an ATF5 deletion mutant that misses the N-terminal activation domain while retains the bZIP domain which is known to be responsible for ATF5 dimerization [26, 31–33]. Immunoblotting analysis showed that WT ATF5 but not dnATF5 interacted with IE86 (Figure 4B). Interaction between endogenous ATF5 and IE86 was further demonstrated in immunofluorescence analysis which showed that the staining patterns of two proteins partially overlapped in the nucleus (Figure 4C). Together, these results indicated that ATF5 Pro-rich domain interacts with IE86.

**Figure 5:**
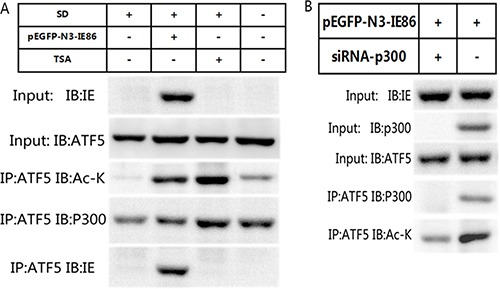
IE86 acetylates ATF5 at K29 through p300 (**A**) IE86 overexpression enhances acetylation of ATF5.U87 cells were transiently transfected with or without IE86 constructs and followed with or without serum deprived and TSA treatment. Anti-ATF5 immunoprecipitates from cells were immunoblotted with indicated antibodies. (**B**) ATF5 acetylation induced by IE86 is p300-dependent. U87 cells were transfected with IE86 constructs and with or without shRNA against p300. Anti-ATF5 immunoprecipitates from cells were immunoblotted with indicated antibodies.

It has already been shown that IE86 appears to act as a multimode transcription factor and it can interact directly with histone acetyltransferases [19, 34]. In order to investigate whether ATF5 is acetylated by interacting with IE86, we transfected U87 with a construct expressing IE86 and cells were maintained with or without serum. Figure 5A showed that the connection between ATF5 and p300 was broken 48 h after serum deprivation (SD) and ATF5 acetylation level was descended. Whereas the expression of IE86 or trichostatin A(TSA), a histone deacetylase (HDAC) inhibitor treatment enhanced the association and acetylation of ATF5 interrupted by SD and rescued cells from programmed cell death evoked by SD. But the depletion of p300 by siRNAs abrogated the ATF5 acetylation induced by IE86 overexpression after serum deprivation (Figure 5B). These results suggest that ATF5 acetylation induced by IE86 is subject to p300-dependent manner.

**Figure 6:**
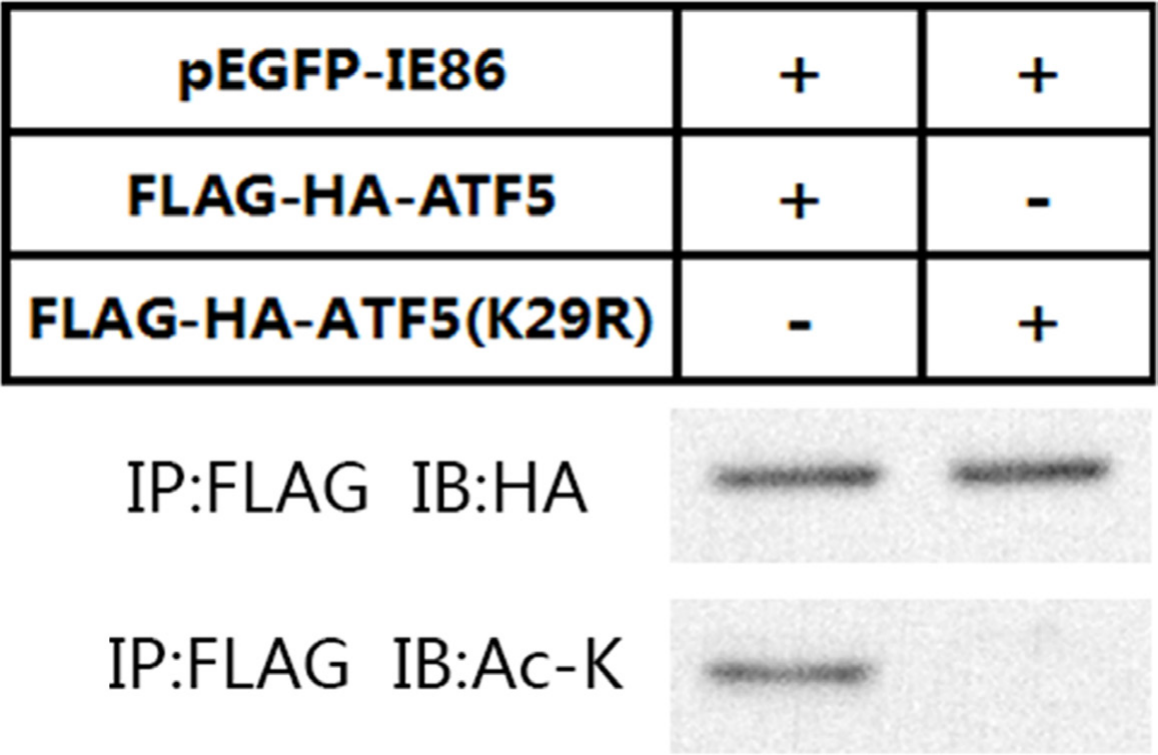
WT ATF5 but not ATF5(K29R) was acetylated by p300 Anti-FLAG immunoprecipitates from C6 cells transiently transfected with indicated constructs were immunoblotted with antibodies against HA or Ac-K.

P300 can acetylate protein substrates at a Gly-Lys (GK) consensus motif. Our previous work has demonstrated an amino acid GK motif (28-GK-29) in the ATF5 is the specific acetylation site of p300 [26]. To exam whether the K29 in ATF5 GK motif is the acetylation site of IE86 as well, we transfected IE86 plasmid and wild-type (WT) ATF5 or ATF5 (K29R) to C6 cells, in which K29 was mutated into arginine. Immunoblotting analysis displayed that WT ATF5 but not ATF5 (K29R) acetylated by IE86 (Figure 6). These results show that ATF5 K29 is a specific target for HCMV IE86-induced acetylation.

### IE expression promoted tumor growth of glioma cells in nude mice

**Figure 7:**
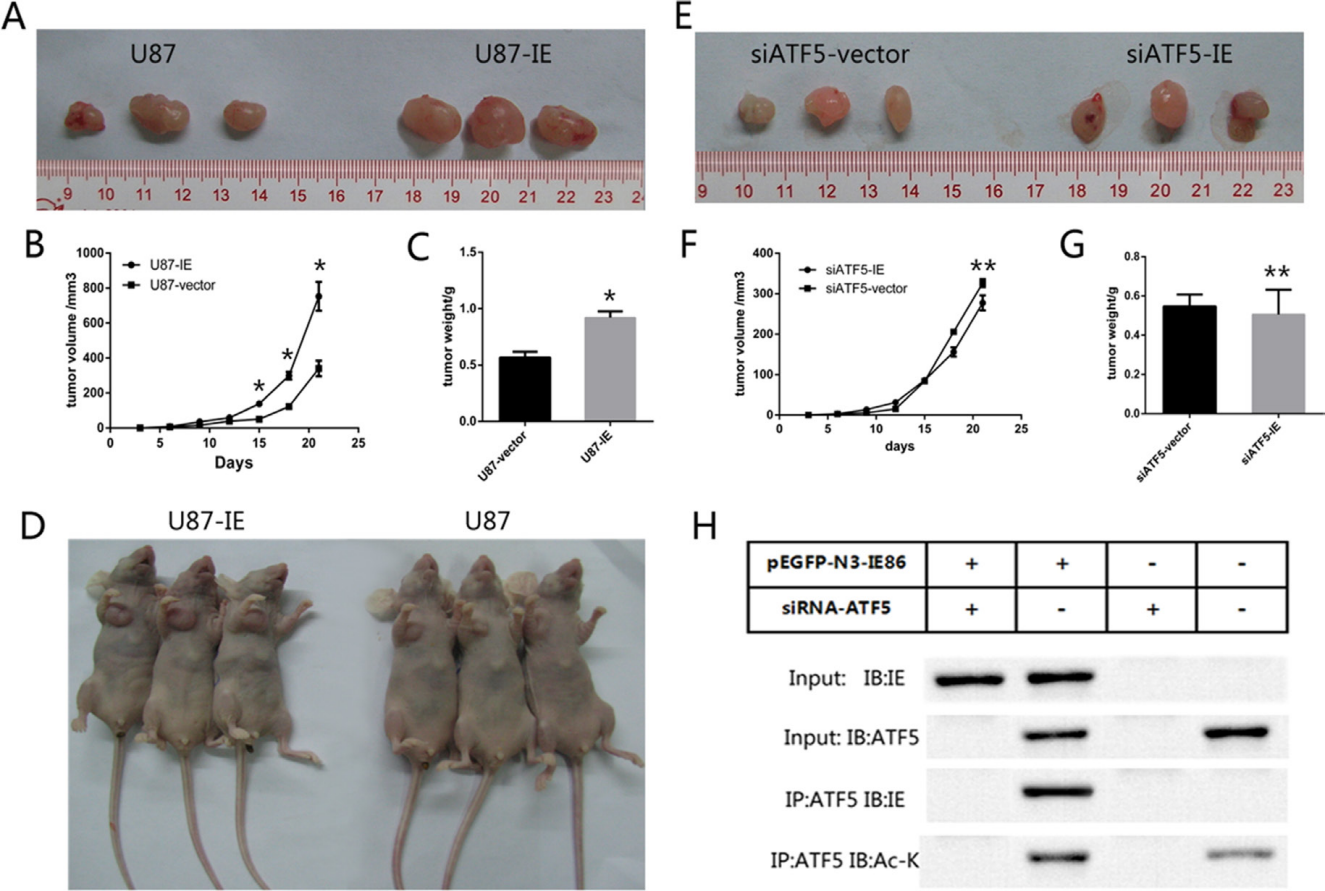
IE86 promoted tumor growth of GBM cells in nude mice (**A** and **D**) U87 cells transfected with IE86 or a vector were injected subcutaneously into the armpit of the nude mice. The mice and tumor were exhibited. (**B**) Growth curve of tumor volumes from the IE86 or vector groups. (**C**) Comparing of tumor weights from the IE86 or vector groups (**E)**. siATF5-U87 cells transfected with IE86 or a vector were injected subcutaneously into the armpit of the nude mice. (**F**) Growth curve of tumor volumes from the siATF5-IE86 or siATF5-vector groups (**G**). Comparing of tumor weights from the siATF5-IE86 or siATF5-vector groups (**H**). Anti-ATF5 immunoprecipitates from cells were immunoblotted with indicated antibodies in xenograft tumors. Groups are divided as indicated. Each data point represents the mean ± SEM of 3 tumors from intratumoral model *P < 0.05 vs vector; **P > 0.05 vs vector.

Our *in vitro* results showed that IE86 could enhance tumor survivability. To expand our findings to an *in vivo* model and to further confirm the correlation of *in vitro* results with the effects of IE expression on U87 cells proliferation *in vivo*, we examined the tumor growth in nude mice. Treatment cell groups were divided as indicated and cells were subcutaneously inoculated into the armpits of the mice. During the three weeks, tumors of IE overexpression group grew much larger and faster than vector transfected group (Figure 7). At the endpoint, the tumor volume and weight of vector group was significantly smaller than that of IE group (Figure 7). While in ATF5 interference groups, subcutaneous xenograft tumors did not grew much larger and faster with the HCMV IE overexpression (Figure 7). To further confirm the function of ATF5 in IE86-driven glioma tumor growth *in vivo*, the xenograft tumors were cut and immunoprecipitation analysis were carried out to show the interaction between IE86 and ATF5 and the acetylation level of ATF5. Immunoblotting analysis showed that ATF5 interacted with IE86 and IE86 overexpression enhanced the acetylation level of ATF5 *in vivo*.

## DISCUSSION

To transform a cell, cytopathic effects and viral replication must be reduced or eliminated in viral infection. These conditions should ring a bell that transformation is a form of persistent infection. One feature of herpesvirus family is persistent infection and this may play a causal role in oncogenesis such as Epstein Barr virus (EBV). HCMV is not recognized as an oncogenic virus, however cells respond to HCMV infection by inducing signaling pathways that ultimately lead to promote cell survival. Recent researches have discovered a high frequency of HCMV in malignancies tumor such as malignant glioma, colon cancer, Kaposi's sarcoma, cervical carcinoma. They revealed the HCMV infection disrupt apoptosis, cell cycle, angiogenesis, cell invasion and the host immune response to catalyze the oncogenic process [35–37]. Our study investigated ATF5-mediated survival mechanism which is essential in malignant glioma genesis is posttranslational regulated by HCMV and revealed a new important mechanism that how does the HCMV infection enhance cancer cell survival in glioma. In this study, we highlighted the relationship of two important regulator proteins, IE and ATF5, in regulating the glioma cells survival under HCMV infection. We found that 1) ATF5 and IE antigens are expressed in different histological types of gliomas but not in non-cancer samples; 2) HCMV infection or IE protein expression enhance glioma cells survivability subject to apoptosis induced by serum deprivation and promote tumor growth of glioma cells *in vivo* via regulating ATF5 expression and post-translational modification; 3) IE protein can interact with N-terminal Pro-rich domain of ATF5 and promote ATF5 acetylation through P300. Our findings confirm the existence of HCMV infection and ATF5 expression in gliomas and reveal an essential role of HCMV IE protein in posttranslational regulation of ATF5 that mediates the unique function of IE in promoting glioma cell survival.

Although there is no exact evidence of on nerve cells transformation after HCNV infection the specific detection of low levels HCMV in human gliomas raises the possibility that this latent infection could enhance the growth or survival of glioma On the other side glioma cells provide a genetic surroundings with features of disturbances signaling pathways, transcriptional factors and tumor suppressor proteins, that enables HCMV to exert its modulatory potential in tumor cells but not in normal cells [36].

In HCMV infected cells, virus protein expressed in three serial stages: immediate-early (IE) which activate early genes necessary for replication; early (E) proteins; and late (L) proteins which are constructive components of the virus. Of this group of genes, the two genetic units in the major IE region designated IE1 and IE2 have been studied in greatest detail. The IE2 gene encodes an essential regulator IE86 protein which is a homodimer with critical functional domains located primarily toward the carboxyl end of the viral protein [38–40]. IE86 has the ability to form a complex with several cellular regulators, including the TATA box-binding protein (TBP), TFIIB, Sp1,Tef-1, c-Jun, JunB, p53, and P300 [19, 38–42]. The IE86 protein is reported to inhibit the induction of apoptosis by TNF-α [43]. The cellular transcription factor CREB is also involved in mediating transactivation by the IE86 protein [44].

Our previous work have confirmed that ATF5 is highly expressed in glioma cells but not in normal brain tissues. As a new member of ATF/CREB family ATf5 can promote cancer cells survival specifically [28, 29]. Inhibition of ATF5 activity, using RNAi or a dominant negative form of ATF5, kills human and rat glioblastoma cells but does not affect normal cells surrounding the tumor, indicating ATF5 is selectively essential for the survival of glioblastoma cells [24, 45]. Therefore, it is reasonable to suppose that the cell survival promoting caused by HCMV infection may be related with ATF5. We showed that HCMV infection or IE expression increase ATF5 expression after serum deprivation but not in a very significantly level. There should be a posttranslational modification after that. The E1A binding protein P300 is transcriptional coactivator protein that could bind to the proline-rich domains of several proteins such as p53 and regulates gene expression through acetylation of nonhistone substrates [46, 47]. The N-terminal region of ATF5 containing about 200 amino acids in which prolines are more than 25% is both rigid and uniquely structured due to high proportion of prolines. Our previous studies showed that P300 binds to and acetylates ATF5 at lysine-29 (K29), which in turn enhances the interaction between ATF5 and p300. ATF5/p300 complex binds to the ATF5 response element (ARE) region of the Egr-1 promoter which enhances cell proliferation and survival [26]. In several instances, apoptosis induced by growth factor deprivation when deprived of serum has been shown to be transcription-dependent and is associated with marked changes in the gene expression profile [48]. We found that the interaction between ATF5 and p300 was interrupted and the acetylation level of ATF5 was also decreased 24 h after serum deprivation. But the overexpression of IE86 protein increased the ATF5 acetylation raising the possibility that IE plays a role in regulation of cell proliferation and survival mediated by acetylated ATF5. Immunoblotting of the immunoprecipitated IE with an anti-ATF5 antibody from C6 cell transfected with a construct expressing GFP-ATF5 or GFP-dnATF5 showed that ATF5 but not dnATF5 associated with IE, indicating that IE specifically interacts with the N-terminal proline-rich domain of ATF5.

Previous studies had shown that both IE and ATF5 are required for survival of transformed cells and share several cellular functions [22, 24, 43, 49–50]. However, their functions do not seem to overlap in cancer cell. In conclusion, our study confirms the existence of HCMV in gliomas and illustrates an ATF5-mediated central role of IE protein regulating glioma cells proliferation and survival in HCMV infected U87 glioma cells. Here we showed that HCMV IE protein does form complexes with endogenous ATF5, which in turn enhances the acetylation of ATF5 that promoting glioma cell survival. These findings reveal that the new IE/ATF5-regulated oncomodulatory mechanism is critically involved in HCMV infected glioma malignant phenotype and this may be a potential therapeutic target.

## MATERIALS AND METHODS

### Immunohistochemistry

The paraffin-embedded human glioma sections specimens were provided by the Department of Pathology of the Affiliated Hospital of Qingdao University Medical College. Paraffin was removed by heating sections at 60°C for 1–2 h, followed by 3 incubations in 100% xylene for 5 min each. Subsequent incubations were in 100, 75, 50 and 0% ethanol for 5 min each. Specimens were then microwaved in 1litre of a sodium citrate buffer (pH = 6) for 45 min for heat-mediated antigen retrieval. A 1% solution of hydrogen peroxide in methanol was used to block endogenous peroxidase activity before transferring the sections into phosphate-buffered saline (PBS) (pH = 7.2). The slides were incubated at room temperature for 1 h with primary antibody(anti-ATF5,anti-IE) at an appropriate dilution (1:200). Peroxidase activity was developed in 0.5% (vol/vol) 3,3′-diaminobenzidine hydrochloride in PBS containing 0.03% hydrogen peroxide for 2 min. then slides were rinsed in tap water, dehydrated, placed in xylene, and mounted at RT. Between each step, slides needed to be washed 3 times (3 min in PBS). The same protocol was performed in the absence of the primary antibody, to show the absence of nonspecific staining in each experiment. Five non-cancer brain tissue samples from astrocyte hyperplasia patients were used as a negative control.

The positive reaction is visible as brown particles. We counted 100 cells in 5 random fields (×400 magnification) and calculated the percentage of positive cells. All slides were evaluated for immunostaining without any knowledge of the clinical outcome or other clinicopathological data. Studies with patient-derived tumors were conducted at Ethics Committee of Qingdao University (Qingdao, China) with written informed consent from each patient.

### Immunofluorescent staining of glioma specimens

Tissue sections of 5-μm thickness were cut from frozen glioblastoma specimens and processed for immunofluorescence. Sections were immunostained with rabbit anti-ATF5 (Abcam, ab60126) and mouse anti-IE (Virostat-Inc, 0841) antibodies and then stained with anti-rabbit IgG conjugated with CY3 (boster, BA1032), and anti-mouse IgG conjugated with FITC (boster, BA1101). Following repeated washes in PBS, nuclei were counterstained with Hoechst dye. The images were obtained using Olympus FV1200 fluorescencemicroscope.

### Cell culture and viruses

U87MG cells were grown in MEM medium (HyClone) supplemented with 10% fetal bovine serum (FBS) (HyClone). For serum deprivation, cells (24 h after transfection if transfected cells were used) were washed with PBS (140 mM NaCl, 2.7 mM KCl, 10 mM Na_2_HPO_4_, and 1.8 mM KH_2_PO_4_, pH 7.4.) and maintained in serum free MEM medium. For drug treatment, trichostatin A (TSA) was added directly to the cell culture (24 h after transfection if transfected cells were used) to a final concentration of as specified. HCMV AD169 (kindly provided by France Pasteur Laboratory and expanded in our laboratory) was tittered by plaque titration in human embryonic lung fibroblast (HELF) cell and expressed as the number of plaque-forming units (PFU) per milliliter. Viruses stored at −80°C. U87MG cells were infected at an approximate multiplicity of infection (MOI) of 10. After 12 h the dish was washed five times and the growth medium was replaced by serum-free medium or indicated medium.

### Transfection and retrovirus infection

Plasmid pEGFP/IE86, pLeGFP-C1-FLAG-ATF5, pLeGFP-C1-FLAG-dnATF5 and was constructed as previously described [32, 51–52]. All plasmid preparations were propagated in Escherichia coli DH5α and purified using the Endo-Free Plasmid Maxi kit (Qiagen). siRNA sequences (CCTGTCTC) targeting P300 were synthesized by Genechem (Shanghai, China). siRNA oligomer were transfected into cells. siRNA sequences (GCGAGATCCAGTACGTCAA) targeting human ATF5 (GenBank accession no: NM 012068), and negative control lentivirus vectors with a scrambled non-targeting shRNA sequence were synthesized by Genechem (Shanghai, China) and cloned into self-inactivating lentivirus vectors: GV113 (Genechem). Cell transfections were carried out using Lipofectamine 2000 (Invitrogen) according to the manufacturer's instructions. Stable cell lines were selected in 400 μg/ml of G418 (Clontech).

### Quantitative real-time reverse transcription PCR

Total cellular RNAs were extracted using Trizol reagent (Invitrogen) following the manufacture's instruction. Two micrograms of total RNA was used as a template for cDNA synthesis using first strand cDNA synthesis kit (Fermentas). Quantitative real-time reverse transcription PCR (qRT-PCR) were carried out using an iCycler IQ system (Biorad Laboratories, Hercules, California,USA) with SYBR Green Master mix (Bio-Rad, USA). The amount of mRNA was normalized to the endogenous reference β-actin and expressed as n-fold mRNA levels relative to the untreated control. Primers for ATF5 detection were 5′- AAGTCGGCGGCTCTGAGGTA -3′ as forward and 5′-GGACTCTGCCCGTTCCTTCA-3′ as reverse; Primers 5′-GCGCAATATCATGAAAGATAAGAACA-3′ and 5′-GATTGGTGTTGCGGAACATG-3′ for IE2; 5′-TGG AACGGTGAAGGTGACAG-3′ and 5′-GGCTTTTA GGATGGCAAGGG-3′ were used for detection of β-actin as internal control. All real-time PCR products were visualised on an agarose gel containing ethidium bromide to confirm the correct amplicon size.

### Immunoprecipitations and Western blot analysis

For immunoprecipitations, cells grown on culture plates were rinsed with ice-cold phosphate-buffered saline (PBS). Cellular proteins were extracted using the Pierce Classic IP kit according to manufacturer's instructions. The separated immune complexes were denatured at 95°C followed by SDS-PAGE and Western blotting using each antibody. For immunoblotting analysis, cells were washed with ice-cold PBS and lysed in RIPA lysis buffer (10 mM Tris-HCl, pH 7.4, 150 mM NaCl, 1% NP-40, 2 mM PMSF, 0.05 mM pepstatin A, 0.2 mM leupeptin). The lysates were centrifuged at 12000 rpm for 10 minutes. Equal amounts of protein (50 μg) were boiled in reducing SDS loading buffer. Samples were subjected to 10% SDS-polyacrylamide gel, and then transferred onto an Immobilon PVDF membrance (Millipore). After blocked with 5% defatted milk for 1 hour at room temperature, the membranes were incubated with antibody overnight at 4°C, washed, and then incubated with secondary antibodies conjugated to horseradish peroxidase. Immunoreactive proteins were detected by using the ECL western blotting detection system, according to the manufacturer's instructions. Antibodies used were anti-ATF5 (Abcam), anti-P300 (Santa Cruz), anti-IE (Virostat), anti-FLAG (Stratagene) and anti-β-actin (Santa Cruz).

### Flow cytometry analysis of apoptosis

Cells were grown to confluency in a 6-well plate. For cell death analysis, gating was adjusted using 7-aminoactinomycin D (7-AAD) staining with dot plots displaying FL3-7-AAD on the y-axis and FL2- annexin V-PE on the x-axis and 5,000 events were collected for each sample. The cells were trypsinized in DMEM, collected by centrifugation, and then cells were treated with both 7-AAD and annexin V-PE for 15 min. Then fluorescence flow cytometric analyses of apoptosis were performed using a Guava EasyCyte Mini instrument (MILLIPORE Guava technologies).

### Immunofluorescence (IF) analysis of glioma cells

U87 and shATF5-U87 cells were maintained in serum free medium and were trypsinized and replated onto glass coverslips in 12-well tissue culture plates. After infection, cells on the glass coverslips were first fixed with buffered 4% paraformaldehyde, pH 7.4, for 30 min at room temperature and then permeabilized with 0.1 M PBS containing 0.2% Triton X-100 for 30 min and thereafter preincubated with 5% normal goat serum for 30 min prior to incubation with the primary antibodies at 4°C for overnight. The primary antibodies employed were against the antigens ATF5 (diluted 1:1000; Abcam) and immediate-early (IE) protein (diluted 1:100; Virostat-Inc). After thorough rinsing with 0.1 M PBS, slides were incubated with the secondary antibody conjugated CY3 (diluted 1:500; boster, BA1032) or FITC (diluted 1:200; boster, BA1101). Following repeated washes in PBS, nuclei were counterstained with Hoechst dye. To ensure antibody specificity, negative control slides were processed in the same manner, but omitting the primary antibodies. The images were obtained using Olympus FV1200 fluorescencemicroscope.

### Mouse xenograft model

All animal experiments were conducted in accordance with the China animal welfare law and approved by local authorities. The athymic BALB/c nude mice (female) weighing 16–18 g were obtained from the Institute of Laboratory Animal Science, Chinese Academy of Medical Sciences. The mice were bred in laminar flow cabinets under specific pathogen-free conditions and handled according to the policies and standards of Laboratory Animal Care in China. U87 cells were overexpressed IE86 48 h before inoculating and maintained in serum free MEM medium. Tumor cells (3 × 10^6^) in 100 μl of PBS were inoculated s.c. into the armpits of the mice. Mice were weighed and tumors were measured with external calipers every 3d and each tumor volume in cubic millimeters was calculated by the following formula: V = 0.5 × D × d^2^ (V, volume; D, longitudinal diameter; d, latitudinal diameter). After 3 weeks, mice were sacrificed by cervical dislocation, xenografts were resected and weighted. Statistical evaluation of differences between groups was performed using 2-tailed student *t* test analysis with unequal variance. *P* < 0.05 was considered significant.

### Statistical analyses

Determination of statistical significance was carried out with two-tailed Student's *t*-test between two groups and ANOVA test for the result of immunohistochemistry. In addition, differences in positivity among the tumor and non-tumor tissues were determined in the IHC slides in a subset of samples (*n* = 56; 25 GBM, 10 anaplastic, 18 low-grade glioma, and 3 non-cancer samples) by scoring 100 cells in 5 independent areas. Analyses comparing every two groups were performed with q-test. Further, the correlation between IE86 and ATF5 immunoreactivity was examined with the Spearman's correlation coefficient. All experiments with statistical analyses were performed at least three times, and data were expressed as the means ± standard deviation (SD). A *p*-value of < 0.05 was considered to be statistically significant. These analyses were performed using PASW 18.0 software (SPSS Inc., Chicago, IL, USA).

## SUPPLEMENTARY MATERIALS FIGURES



## Abbreviations

HCMV: Human cytomegalovirus
IE86: immediate-early protein 86
ie2: immediate-early 2 gene
ATF5: activating transcription factor 5
CBP: CREB-binding protein
SD: serum deprivation
K29: lysine-29.
