# Supplementary material for: Human cytomegalovirus immediate-early protein promotes survival of glioma cells through interacting and acetylating ATF5
Source: Oncotarget. 2017 Apr 17;8(19):32157–70. doi: 10.18632/oncotarget.17150 (PMC5458275; doi:10.18632/oncotarget.17150)
Supplement: Supplementary file 1 [file oncotarget-08-32157-s001.pdf]

Human cytomegalovirus immediate-early protein promotes survival of glioma cells through interacting and acetylating ATF5

Supplementary Materials

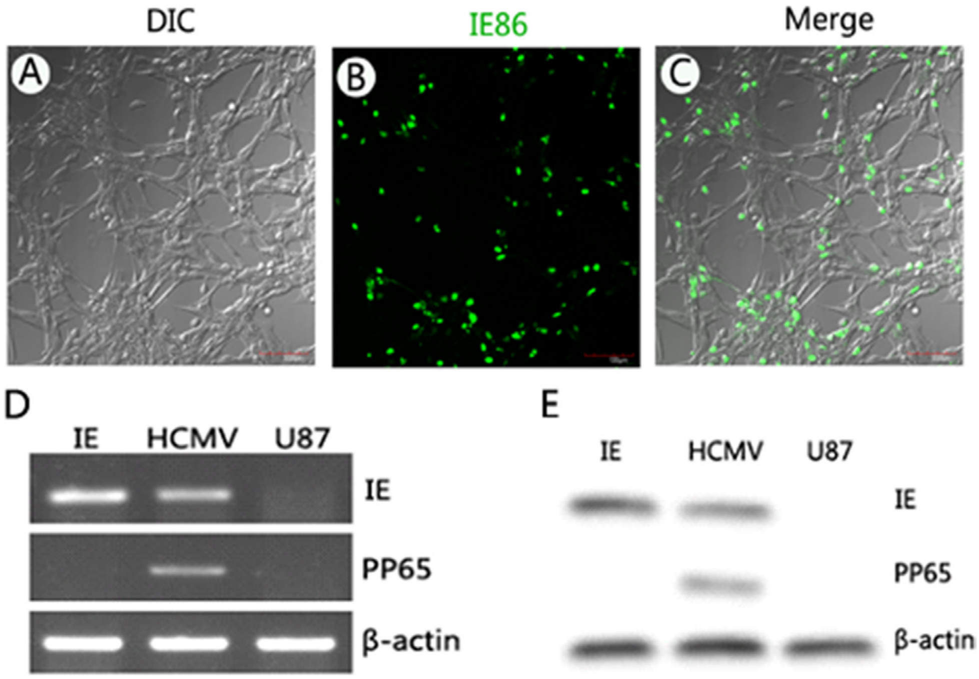

Supplementary Figure 1: Expression of HCMV IE86 and PP65 in U87 human glioblastoma cells. (**A–C**) immunofluorescence detecting of IE86 in U87 cells transfected with plasmids encoding IE. (**D**) RT-PCR analysis monitoring IE86 and PP65 expression in U87 cells after HCMV infection or IE plasmids transfection. (**E**) western-blot analysis IE86 and PP65 protein expression in U87 cells after HCMV infection or IE plasmids transfection.

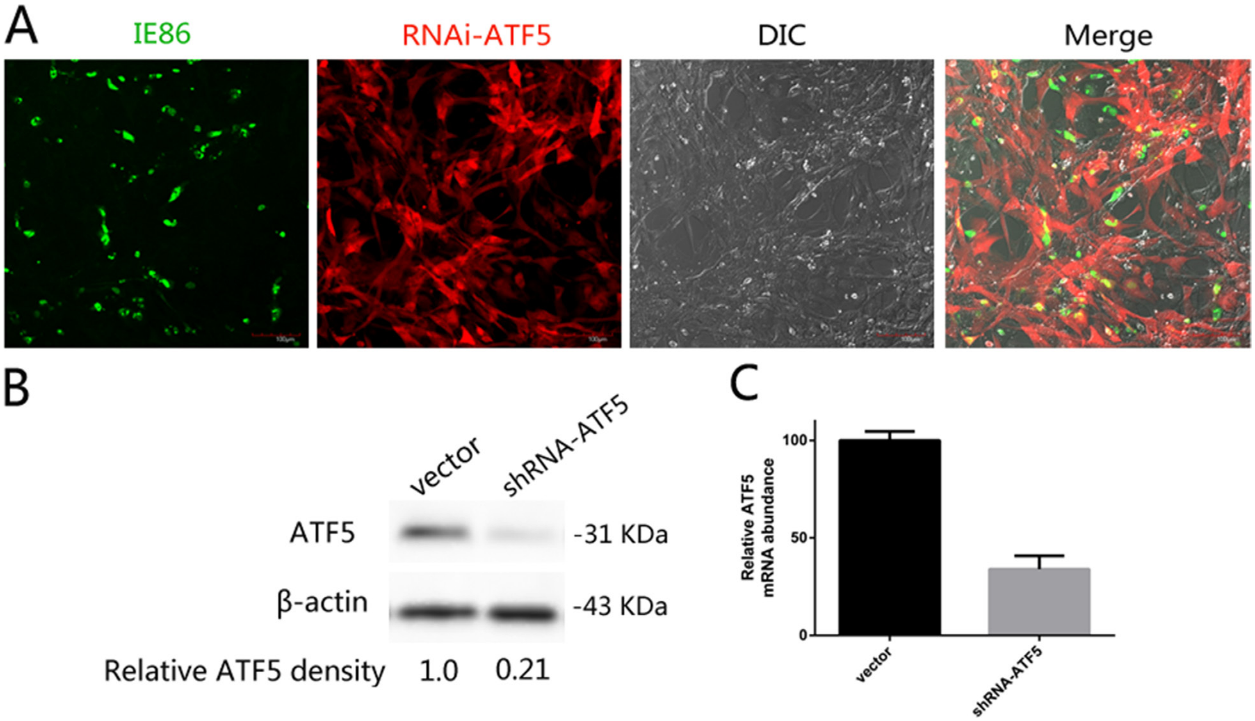

Supplementary Figure 2: Establishment of lentivirus delivery RNAi-ATF5 U87 cell lines. (**A**) immunofluorescence detecting of RNAi ATF5 plasmid lentivirus infection(red) and IE86 expression(green) in U87 cells. (**B**) western-blot analysis monitoring ATF5 expression in U87 cells stably transfected with plasmids encoding siRNA ATF5 sequences. (**C**) qRT-PCR analysis monitoring ATF5 expression in U87 cells stably transfected with plasmids encoding siRNA ATF5 sequences.
